# Properties of Tumor Spheroid Growth Exhibited by Simple Mathematical Models

**DOI:** 10.3389/fonc.2013.00051

**Published:** 2013-03-15

**Authors:** Dorothy I. Wallace, Xinyue Guo

**Affiliations:** ^1^Department of Mathematics, Dartmouth CollegeHanover, NH, USA

**Keywords:** tumor spheroid, mathematical oncology, mathematical biology, tumor models, necrosis, quiescence, tumor simulation

## Abstract

Solid tumors, whether *in vitro* or *in vivo*, are not an undifferentiated mass of cells. They include necrotic regions, regions of cells that are in a quiescent state (either slowly growing or not growing at all), and regions where cells proliferate rapidly. The decision of a cell to become quiescent or proliferating is thought to depend on both nutrient and oxygen availability and on the presence of tumor necrosis factor, a substance produced by necrotic cells that somehow inhibits the further growth of the tumor. Several different models have been suggested for the basic growth rate of *in vitro* tumor spheroids, and several different mechanisms are possible by which tumor necrosis factor might halt growth. The models predict the trajectory of growth for a virtual tumor, including proportions of the various components during its time evolution. In this paper we look at a range of hypotheses about basic rates tumor growth and the role of tumor necrotic factor, and determine what possible tumor growth patterns follow from each of twenty-five reasonable models. Proliferating, quiescent and necrotic cells are included, along with tumor necrosis factor as a potential inhibitor of growth in the proliferating pool and two way exchange between the quiescent and proliferating pools. We show that a range of observed qualitative properties of *in vitro* tumor spheroids at equilibrium are exhibited by one particular simple mathematical model, and discuss implications of this model for tumor growth.

## 1 Introduction

Tumor spheroids cultured *in vitro* play an important role in cancer research. Various authors have pointed out that spheroids are a better representation of many types of *in vivo* tumors than monolayer culture, and less expensive than *in vivo* studies, as described by Santini et al. (2000) and Hirschhaeuser et al. (2010).

Spheroid growth is observed to cease spontaneously, with a characteristic long term anatomy and a variety of terminal volumes for any given cell line. At the earliest stage the spheroid may be an undifferentiated mass of proliferating tumor cells. At an intermediate stage the proliferating cells form a shell around the outside of the spheroid and the inner core will consist of live cells that are not actively dividing, which we will refer to as “quiescent.” These have been observed in tumor spheroids via imaging techniques (Sherar et al., 1987) and through isolation and staining (Preisler et al., 1977; Bauer et al., 1982). At later stages the inner core of the spheroid will be necrotic tissue, surrounded by a shell of quiescent cells, and an outermost layer of live, proliferating cells (Folkman and Hochberg, 1973; Sherar et al., 1987). Proliferating cells are the target of most cancer therapies. The quiescent cell population has been implicated as a population resistant to some of these therapies, playing an important role in tumor regrowth (Potmesil and Goldfeder, 1980; Kallman et al., 1982).

Numerous models for tumor spheroids, quiescence, and necrosis, are in the literature, and these exhibit the observed phenomena to greater or lesser degree. Models range from extremely complex to simple with an enormous range in between. Simple models only attempt to match total tumor size (Marusic et al., 1994; Demidenko, 2006). These find a reasonably good match with logistic and Gompertz equations, which postulate a known bound on spheroid size.

The necrotic core is a feature of all but the simplest models. Tumor necrosis factor has been implicated as the cause of the eventual cessation of growth in spheroids (Freyer, 1988). Greenspan (1972) is possibly the earliest such model, employing numerous simplifying assumptions to arrive at differential equations that can be solved explicitly. Menchon and Condat (2008, 2009) come to the conclusion, based on mathematical models, that some inhibitory factor is necessary for growth cessation in spheroids. This has been a general observation for mathematical models that do not include an *a priori* known bound for the size of the spheroid in the governing equations, as is the case for logistic or Gompertz models.

Some models take diffusion of nutrients into account, producing the characteristic distribution of proliferating, quiescent and necrotic cells, relying on a variation of the diffusion equation and parameters for a variety of nutrients (Venkatasubramanian et al., 2006). An early example is by Gyllenberg and Webb (1990), whose model includes both quiescence and necrosis to arrive at a growth trajectory that resembles the observed Gompertz curve, but which drives the proliferating cell population to zero. To this scenario, some authors add cellular motion (Stein et al., 2007), a consideration of the forces that determine the shape of the tumor mass (Frieboes et al., 2006), and mechanisms for the onset of necrosis (Menchon and Condat, 2008). Others achieve the similar structure through a combination of models at different level of structure (Jiang et al., 2005).

A series of models by Adam (1986, 1987a,b) dating back to the 1980s uses one dimensional diffusion with nutrient source and a time independent source of unspecified mitotic inhibitor responding to a switch. The continuously growing necrotic core observed in experiment suggests that the production of mitotic inhibitor is neither time independent nor switched discontinuously. Adam notes in his third paper that the necrotic core would have to be taken into account in future models, such as those presented in this paper, in order to match the observations in Folkman et al. (Folkman and Hochberg, 1973; Adam, 1987b). The more sophisticated treatment in Maggelakis and Adam (1990) yields the observed Gompertz curve but does this paper does not give information about the composition of the tumor at equilibrium. A similar series of models by McElwain and coauthors (McElwain and Ponzo, 1977; McElwain, 1978; McElwain and Morris, 1978) consider the diffusion process in a spheroid in detail.

The advantage of complex models is that they produce a range of outcomes, some of which are similar to observed growth patterns. The disadvantage is that they require knowledge of many specific parameters, some of which are hard to obtain. The models we consider in this paper are similar to the one developed by Landry et al. (1982), which over a limited time agreed with data from Folkman and Hochberg (1973) but did not include a quiescent component and did not have the bounded growth characteristic of tumor spheroids. They are also similar to one proposed by Piantadosi (1985), which includes all three components and places a bound on the reproducing subpopulation. These are some of the older, simpler models in the literature. They do not attempt to explain shape, only quantities of various cell types. They were developed before an understanding of the potential role of tumor necrosis factor, and deserved to be revisited with that role in mind. None of these examples includes the return of quiescent cells to the proliferating pool.

The goal of this paper is to find the simplest possible system of ordinary differential equations that produces the qualitative results observed in Folkman and Hochberg (1973), Sherar et al. (1987), and Freyer (1988), starting from the assumption that proliferating, quiescent and necrotic layers exist. As part of this search, we will rule out a large collection of simple models that, although conceptually reasonable, do not produce results consistent with these three papers. Simple, as interpreted here, means a system of ordinary differential equations that has approximately as many parameters as there are measured quantities. In these equations, diffusion is assumed to be uniform in the proliferating compartment and sufficient to produce growth. The movement of nutrient is not modeled, except as a variation between compartments. The image of a spheroid at equilibrium from the Folkman and Hochberg paper shows a very thin shell of proliferating cells, hardly enough to make the effects of diffusion prominent. Similarly, tumor necrosis factor inhibiting growth of proliferating cells is assumed to reach those cells uniformly. Although clearly a simplification in some respects, such models are desirable as they allow an approximate fit to measured data with the correct range of qualitative behaviors, without requiring knowledge of quantities that are not easily measured and with few enough parameters that the range of possible answers is small.

These papers describe four quantities at moments in time: the tumor size in Folkman and Hochberg (1973) and Freyer (1988), and the amounts of proliferating, quiescent and necrotic cells in Sherar et al. (1987). The basic qualitative results are summarized in the following list.

1. Spheroids, no matter what the initial conditions may be, eventually develop three layers of proliferating, quiescent and necrotic cells (Sherar et al., 1987).
2. Growth of spheroids eventually stops. (Folkman and Hochberg, 1973).
3. When the growth stops, there remains a thin layer of actively proliferating cells at the boundary (Folkman and Hochberg, 1973).
4. The final size of the spheroids is correlated with thickness of the proliferating shell (Freyer, 1988).
5. The final size of the spheroids is correlated with the size at which necrosis begins (Freyer, 1988).
6. The more spheroids in a flask, the smaller the average size when growth ends (Folkman and Hochberg, 1973).

### 1.1 Development of models

The models developed for this study track the dynamics of a three part tumor spheroid growing *in vitro*. The quantities tracked are

1. Proliferating cells, which after time form a concentric shell at the exterior of the spheroid, as observed in numerous imaging studies, including those of Freyer (1988), Folkman and Hochberg (1973), and Sherar et al. (1987). These are exposed to the nutrient solution. They may become quiescent or they may die and be shed from the spheroid. In early stage spheroids the proliferating cells may constitute the entire spheroid and grow at an intrinsic rate. A fraction of proliferating cells may also be shed into the surrounding medium.
2. Quiescent cells, which form a secondary, and usually thicker, shell inside the outer layer of proliferating cells. These arise as proliferating cells pass to a quiescent state. They may return to a proliferating state or experience cell death due to necrosis.
3. Necrotic cells, which form the core of the spheroid. These can be absent in small spheroids or constitute the majority of the spheroid mass in older cultures. They arise as quiescent cells die. Necrotic cells may also undergo dissolution and be removed from the system entirely.
4. Total spheroid size.

The basic system of three non-linear ordinary differential equations coming from items 1 to 3 above is shown in full generality below, with explanations of the individual terms following. Note that 25 variations result from our considerations, each labeled with a number from 1 to 5 and a letter from A to E.

(1) $$\begin{array}{rc} & \frac{dP}{dt}=G\left(P\right)-b_{P,Q}P+c_{Q,P}Q-F\left(P,Q,N\right)-dP \end{array}$$

(2) $$\begin{array}{rcl} & \frac{dQ}{dt}=b_{P,Q}P-c_{Q,P}Q-e_{Q,N}Q+H\left(P,Q,N\right) & \end{array}$$

(3) $$\begin{array}{rcl} & \frac{dN}{dt}=e_{Q,N}Q-mN & \end{array}$$

#### 1.1.1 Growth of proliferating cells, *G*(*P*)

Growth of tumor spheroids is observed to cease. Thus there is some limiting factor on the growth of *P*. However, there is debate about the nature of this factor. A limit can be imposed directly by using logistic or Gompertzian growth for *P*, both of which have similar qualitative properties. However the access of proliferating cells to nutrient is likely to be dependent on the surface area of the spheroid, with any limitation to growth due to other factors such as tumor necrosis. Models 3, 4, and 5 below have different versions of this hypothesis. Yet another alternative is to assume simple exponential growth of P. We look at five variations of the function *G*(*P*). All models include a death rate of proliferating cells, *dP*, that are assumed to be shed into surrounding medium and lost from the model.

1. Model 1 uses a logistic term to limit the growth of *P*: *G*(*P*) = *aP*(1 − *P*) We could have used a Gompertzian model with similar qualitative results.
2. Model 2 assumes exponential growth: *G*(*P*) = *aP*, as in Piantadosi (1985).
3. Model 3 assumes that growth is proportional to the surface area of the spheroid: *G*(*P*) = *a*(*P* + *Q* + *N*)^2*/*3^.
4. Model 4 assumes that growth is jointly proportional to both surface area and volume of P: *G*(*P*) = *aP*(*P* + *Q* + *N*)^2*/*3^.
5. Model 5 uses the surface area of the spheroid as the limiting factor in a logistic growth term: *G*(*P*) = *aP*(1 − *P*(*P* + *Q* + *N*)^−2*/*3^).

#### 1.1.2 Transition from proliferating to quiescent, *b_P,Q_P*, *c_Q,P_Q* and quiescent to necrotic, *e_Q,N_Q*

Tumor spheroids exhibit a layer of quiescent cells (Sherar et al., 1987) which are thought to arise as proliferating cells lose access to nutrients. Similarly, quiescent cells die after sufficient lack of nutrient. In addition, it is known that quiescent cells may become proliferating cells again (Potmesil and Goldfeder, 1980), and so a return loop is included in the model, with a proportion of *Q* returning to the proliferating pool. These terms remain the same across all the models studied here. In *in vivo* tumors, the location of quiescent cells (and also necrotic cells) would depend on the geometry of this access, including the location of blood vessels, and the growth of these classes of cells is difficult to measure. For simplicity we use linear terms to describe this transition. There are two justifications for this. First, if we assume that passage to the quiescent state is a result of the limits of diffusion of nutrients, then as *P* approaches a limiting thickness the amount of proliferating cells transitioning to quiescent is proportional to the surface of the inside of the proliferating shell. Near the limiting thickness surface area is approximately proportional to volume of *P*. Second, whatever rule governs the transition from *P* to *Q* may be expressed as a Taylor series in *P* whose leading term must be the linear one. For both of these reasons, a good first approximation to this process is linear dependence on *P* given by *b_P,Q_P*. Similar arguments may be made for the other terms, *c_Q,P_Q* and *e_Q,N_Q*. Thus we assume that constant proportion of *P* becomes *Q*, and a constant proportion of *Q* dies to become *N*.

#### 1.1.3 The effect of tumor necrosis factor, *F*(*P*, *Q*, *N*) and *H*(*P*, *Q*, *N*)

Extract of necrotic tumors is known to reduce the growth of tumor spheroids (Freyer, 1988), but the mechanism is unclear. It is possible that as quiescent cells become necrotic they release a substance that slows growth of proliferating cells. It is possible that the necrotic cells themselves continue to release such a substance. Finally, it is possible that some substance increases the rate at which proliferating cells become quiescent, and perhaps this is enough to stop growth. We have looked at all of these hypotheses, and summarized them in five cases.

(A) Model A assumes that the proliferation of *P* is slowed when proliferating cells come in contact with substances released as quiescent cells die. *F* is thus proportional to both *P* and the rate of necrosis, *cQ*, so that *F*(*P*, *Q*, *N*) = *fQP*. This term can be interpreted as slower growth or as death and shedding of *P* cells; mathematically it makes no difference. The interesting feature of this model is that the growth reducing effect of necrosis is determined by the size of *Q* and thus is bounded in models where *Q* approaches equilibrium. In this model no extra rate of quiescence is assumed, so *H*(*P*, *Q*, *N*) = 0.
(B) Model B only assumes that the passage of cells from proliferating to quiescent is increased in proportion to the number of proliferating cells. In this model, *F*(*P*, *Q*, *N*) = 0 and *H*(*P*, *Q*, *N*) = *hP*, so there is effectively no tumor necrosis factor that depends on *Q* or *N*.
(C) Model C includes features of both Model A and Model B, so *F*(*P*, *Q*, *N*) = *fQP* and *H*(*P*, *Q*, *N*) = *hP*.
(D) Model D assumes that the proliferation of *P* is slowed when proliferating cells come in contact with substances released by all cells in the necrotic pool. *F* is thus proportional to both *P* and *N*, so that *F*(*P*, *Q*, *N*) = *fNP*. This term can be interpreted as slower growth or as death and shedding of *P* cells; mathematically it makes no difference. However, in models where *P* and *Q* do not go to zero, *N* can get arbitrarily large, unlike in models A and C. This model includes increased passage of proliferating cells to the quiescent pool, so *H*(*P*, *Q*, *N*) = *hP*.
(E) Model E assumes only that the proliferation of *P* is slowed when proliferating cells come in contact with substances released by all cells in the necrotic pool, so *F*(*P*, *Q*, *N*) = *fNP* and *H*(*P*, *Q*, *N*) = 0.

#### 1.1.4 Dissolution of necrotic cells, *mN*

A cell that is dead long enough may dissolve and its contents be removed from the system. Indeed, this may be the very source of substances that retard growth. For our initial numerical experiments, *m* was taken to be zero. In the results section we discuss the various growth patterns that result from these experiments, a few of which have good properties of the *P* and *Q* compartments, but which have constantly increasing values for *N*. This is reasonable because the system is live and dynamic, so there is always some death taking place. Taking *m* to be any positive constant allows *N* to reach equilibrium in these cases. Unless otherwise stated, however, we take *m* = 0.

#### 1.1.5 Constants

Default constants for all runs are *a* = 0.01, *b_P,Q_* = 0.01, *c_Q,P_* = 0.005, *d* = 0.002, *e_Q,N_* = 0.002, *f* = 0.01, *h* = 0.001, *m* = 0. These constants always give tumors that grow, at least initially. Clearly, for each of these models we could choose sufficiently slow growth (or fast death) so that the tumor size decreases, but this is the less interesting case. Note that the constants were chosen for the purposes of comparing models and do not represent any particular cell line or data set. The constants chosen for these runs may all be scaled to a different time frame. A more realistic growth parameter, *a*, would be about 70 times larger than the one we chose here if the time unit is 1 day. Scaling all constants together results in faster growth but keeps equilibrium values the same. Data on how the volumes or cell counts of the *P*, *Q*, and *N* pools change over time for a particular type of spheroid is not available, so it is not possible to infer the constants in the model with any certainty. The results in this paper concern qualitative observations that depend on equilibrium values only, and therefore do not depend strongly on the exact constants chosen, as long as the system arrives at equilibrium.

## 2 Materials and Methods

All twenty-five models were run with default settings using Matlab ODE 45 solver to compare qualitative outcomes. Subsequent comparisons and graphs for all figures in this paper were run on BGDEM software developed by Brian Reed. Adobe Photoshop was used to format all graphs for publication.

The twenty-five models under consideration fall into three broad groups when *m* = 0:

1. Models where *P* and *Q* approach a non-zero equilibrium: 1A, 1B, 1C, 2A, 2C, 3A, 3C, 3D, 3E, 5A, 5C. These models exhibit the basic qualitative behaviors of *P* and *Q* described in the literature.
2. Models in which *P* and *Q* approach zero: 1D, 1E, 2D, 2E, 4A, 4B, 4C, 4D, 4E, 5D, 5E. In these models total tumor growth stops as this occurs, as the growth of *N* stops. Introducing a small value for *m* does not change this behavior. It is possible that other behaviors would appear if *m* were large enough, but those behaviors would then depend on a parameter for which there is, as yet, no estimate.
3. Models in which *P* and *Q* grow without bound: 2B, 3B, 5B.

These results are summarized in Table 1, where * denotes a non-zero equilibrium, 0 denotes cases where *P* and *Q* approach zero, and u denotes unbounded growth.

**Table 1:** **Summary of model behaviors for all cases**.

	1	2	3	4	5
A	*	*	*	0	*
B	*	u	u	0	u
C	*	*	*	0	*
D	0	0	*	0	0
E	0	0	*	0	0

* denotes a non-zero equilibrium, 0 denotes cases where P and Q approach zero, and u denotes unsounded growth.

**Figure 1:**
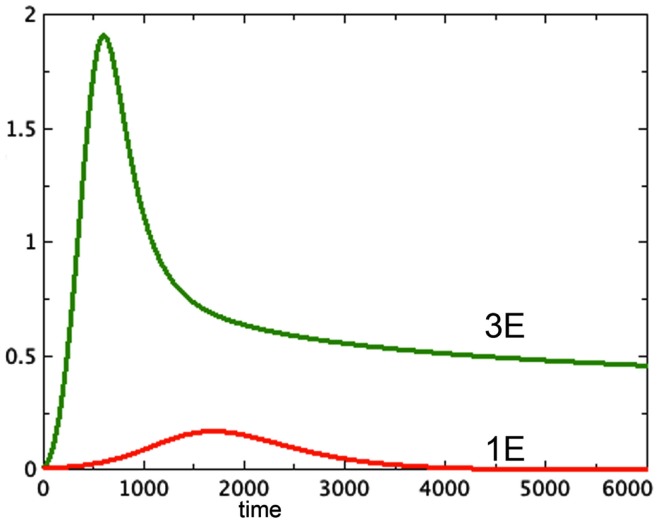
**Proliferating pools from models 3E and 1E compared**. *a* = 0.01, *b_P,Q_* = 0.01, *c_Q,P_* = 0.005, *d* = 0.002, *e_Q,N_* = 0.002, *f* = 0.01, *h* = 0, *P*_0_ = 0.01, *Q*_0_ = 0, *N*_0_ = 0.

A typical run from each of the first two categories is shown in Figure 1.

### 2.1 Some comments on the equations

The equation for *Q*′ is a simple linear relationship between *P* and *Q*. Thus, if *P* reaches an equilibrium, so will *Q* in corresponding proportion, for all twenty-five models. Similarly, for all models *N*′ is positive if *m* = 0, and will continue to increase as long as *Q* (and therefore *P*) is positive. Models for which *P* and *Q* go to non-zero equilibrium will thus still have *N* increasing. However this is a problem that can be easily solved by taking *m* to be any positive number, forcing *N* to an equilibrium. For models in which tumor growth depends on *N* (Models *D* and *E*), this has the effect of creating a non-zero equilibrium in cases that would otherwise have *P* and *Q* going to zero.

Model 3E, which we examine further in this paper, has non-zero equilibrium points given by the following equations.

(4) $$\begin{array}{rc} Q & =\frac{b}{c+e}P \end{array}$$

(5) $$\begin{array}{rcl} N & =\frac{e}{m}Q & \end{array}$$

(6) $$\begin{array}{rcl} 0 & =wX^{4}-vX-u & \end{array}$$

Here *X* = *P*^1*/*3^, *u* = *a*(1 + α + β)^2*/*3^, *v* = (−*b* + *c*β − *d*), *w* = *f*α, $\alpha =\frac{eb}{m\left(c+e\right)},$ $\beta =\frac{b}{\left(c+e\right)}$. The derivative of the right hand side of equation (6) has at most one real root, thus equation (6) has at most two real roots. By DesCartes’ rule of signs (for positive parameters *w*, *v*, *u*) equation (6) has at most one real root. Thus, the non-zero equilibrium, if it exists, is unique.

## 3 Results

### 3.1 Model B

These are the models in which necrotic factor plays no role. Of these, only 1B, which has logistic growth, reaches a non-zero equilibrium for *P* and *Q*, and that equilibrium is independent of initial conditions. The same would hold if we replace the logistic term with a Gompertz equation. The criticism of this model is conceptual. If there is a limit to growth, what is causing it? The limits of diffusion explain uniform spheroids of proliferating cells, but not actual spheroids, which grow to have a necrotic core that does not require nutrient. The thin shell of proliferating cells is within the range of diffusion, and should therefore not be limited in growth. As the spheroid grows, the surface with its thin layer of proliferating cells resembles a plate culture, which is known to grow in an unlimited fashion.

In model 4B, *P* and *Q* go to zero. This gives a spheroid of limited size, but it is dead. Models 2B, 3B, and 5B display unlimited growth.

### 3.2 Models A and C

Models 1A, 1C, 2A, 2C, 3A, 3C, 5A, and 5C all show *P* and *Q* going to a non-zero equilibrium. This equilibrium may be calculated directly from the equations and does not depend on *N*, which continues to grow in these models. Thus, adding extra necrotic factor to these models (by increasing the initial quantity of *N*, for example) will have no effect on the eventual size of the proliferating and quiescent pools. It would be difficult to duplicate the results in Freyer (1988), which display a dependence of spheroid size on various factors related to the quantity *N*, using these models.

### 3.3 Models 3D and 3E

These models represent the best fit with qualitative observations. The growth term reflects the assumption that diffusion is the driving supplier of nutrients. The only difference between these two models is the rate at which proliferating cells become quiescent. In both of these, *P* and *Q* stabilize as *N* continues to grow, but the equilibrium values of *P* and *Q* cannot be deduced from the equations, which depend on *N*. By adjusting the value of *m* to be positive, we can arrange for *N* to arrive at any equilibrium value (depending on the equilibrium value of *Q*).

**Figure 2:**
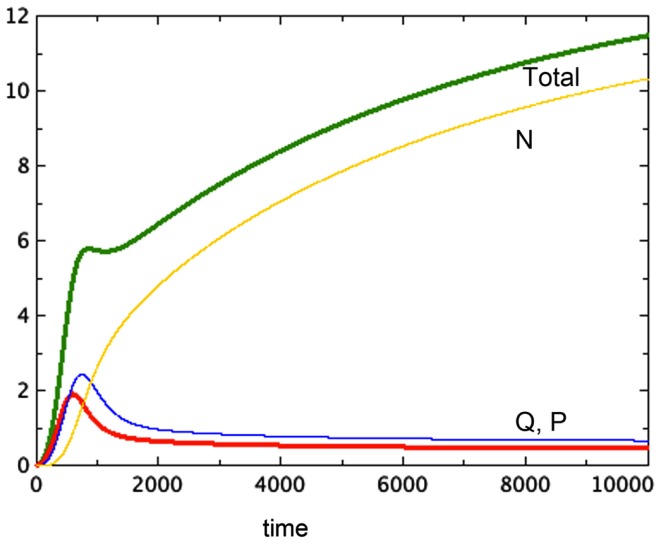
**A typical run of Model 3E**. *a* = 0.01, *b_P,Q_* = 0.01, *c_Q,P_* = 0.005, *d* = 0.002, *e_Q,N_* = 0.002, *f* = 0.01, *h* = 0, *m* = 0.0001, *P*_0_ = 0.01, *Q*_0_ = 0, *N*_0_ = 0.

The hypothesis of growth that is dependent on surface area (Model 3) gives the best representation of experimental data among the various models tested. Model 3E will always include a non-trivial equilibrium for quiescent cells, as observed in Sherar et al. (1987). Furthermore, this model only arrives at equilibrium in the presence of a tumor necrotic factor that depends on the actual quantity of necrosis that has occurred (Models D and E). Finally, the extra feature of faster passage of proliferating cells to quiescent does not play an important role here, with the caveat that only a very simple form of this extra feature was tested. Figure 2 shows a typical run of Model 3E.

### 3.4 A closer look at experimental results

**Figure 3:**
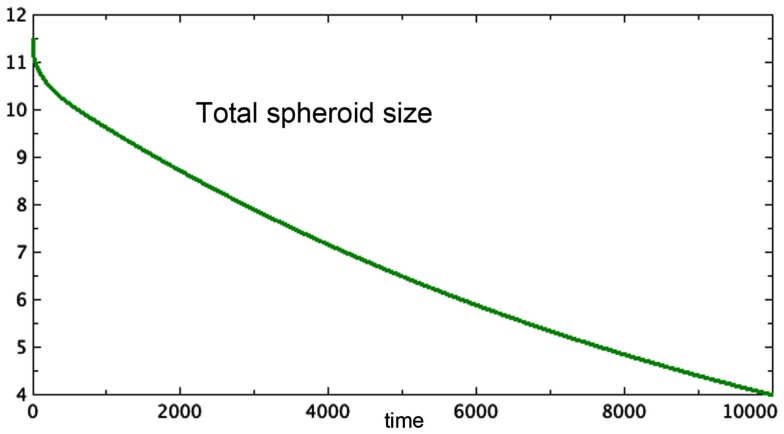
**Approximate terminal values at *t* = 10,000 for the run shown in Figure 2 are starting values in this run of Model 3E**. Only total spheroid size is plotted. The growth rate of *P* was drastically decreased. *a* = 0.0001, *b_P,Q_* = 0.01, *c_Q,P_* = 0.005, *d* = 0.002, *e_Q,N_* = 0.002, *f* = 0.01, *h* = 0, *m* = 0.0001, *P*_0_ = 0.426, *Q*_0_ = 0.681, *N*_0_ = 10.4.

We notice the following phenomena in Model 3E, which mirror the results of Folkman and Hochberg (1973):

1. Figure 2 shows a typical run of Model 3E, displaying the proliferating pool and the total spheroid size. In early stages of growth, the proliferating pool is a large fraction of the spheroid, while at equilibrium the proportion of the tumor accounted for by proliferating cells is much smaller. This phenomenon was observed by Folkman and Hochberg (1973), with proliferating cells being as much as 60% of the spheroid volume in early stages and dropping to 14% at equilibrium.
2. The data displayed in that same paper show a distinctive early overshoot of both the total volume and the proliferating pool, followed by a slight drop as the spheroid approaches equilibrium. That overshoot is also present in Figure 2 of this paper, Model 3E.
3. Figure 3 shows a late stage version of the 3E spheroid, in which the growth rate of proliferating cells is reduced drastically. Note that the growth term for this model is proportional to surface area, whereas the removal of *P* is linear. The two processes are not symmetric. The spheroid volume is seen to drop in a linear fashion. This was observed *in* *vitro* by Folkman and Hochberg when dormant spheroids were exposed continuously to a substance that prevented mitosis.

We now turn to the experiments done by Freyer (1988), in which a variety of cell lines were cultured as spheroids in flasks. Several features of this experiment are particularly important from the modeling standpoint. First, cell lines were cultured separately. Second, flasks were renewed with added nutrient and by removing excess spheroids to ensure a steady supply of nutrient to each spheroid. Third, multiple spheroids of varying sizes were in each flask. Freyer observes that the only parameter of the spheroids that he measured which was positively correlated with saturation size was the thickness of the viable cell rim. From the point of view of our models, this is the statement that the equilibrium values of *P* and total spheroid size at equilibrium were positively correlated. We show one scenario that results in the correlation observed by Freyer.

**Figure 4:**
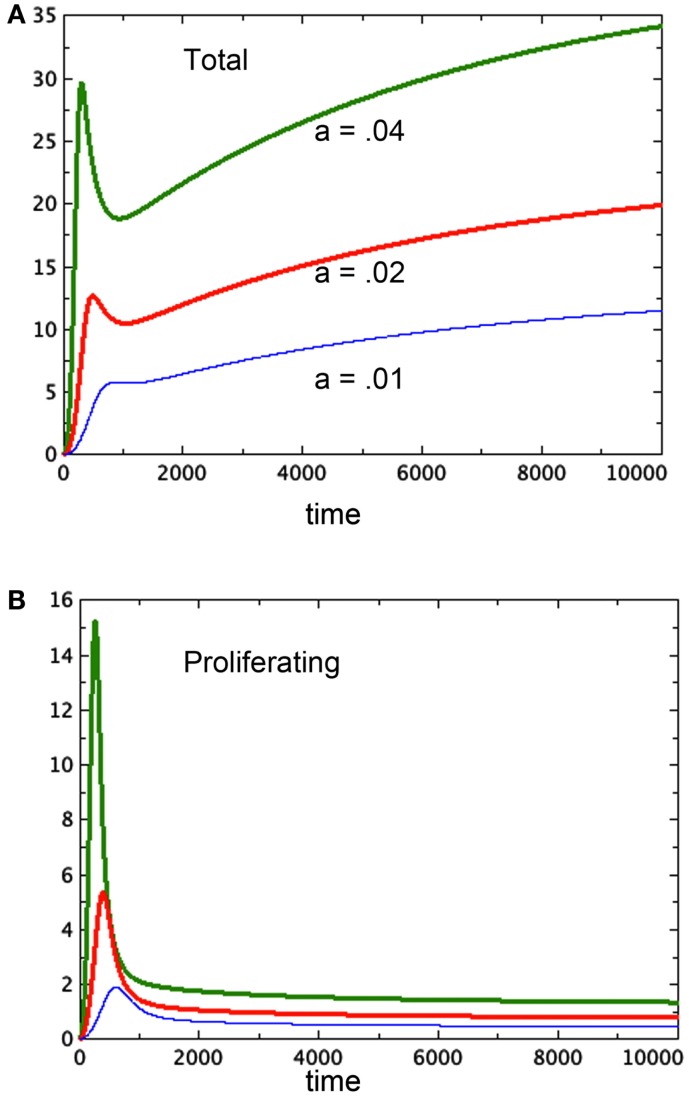
**Three runs of Model 3E with different growth factors as labeled**. **(A)** Shows the total spheroid size and **(B)** shows the proliferating pool. As in Figure 2, *b_P,Q_* = 0.01, *c_Q,P_* = 0.005, *d* = 0.002, *e_Q,N_* = 0.002, *f* = 0.01, *h* = 0, *m* = 0.0001, *P*_0_ = 0.01, *Q*_0_ = 0, *N*_0_ = 0.

Freyer proposes that the variation in final size could be the result of variation in parameters associated to cell growth or decay. We can model this as a variation in the growth parameter *a*. In Figures 4A,B we compare three runs of revised model 3E at *a* = 0.04, 0.02, 0.01 respectively. The figure shows that the equilibrium values of both the spheroid size and the size of the proliferating pool are positively correlated. Further, one can compare the equilibrium values of the proliferating pool with the equilibrium values of the spheroid size raised to the 2/3 power, which correlates with surface area of the tumor. This ratio increases with tumor size in the example shown, indicating a thicker rim of proliferating cells in larger tumors, as observed in experiment. For the runs pictured, the ratios are 0.11, 0.096, and 0.067 respectively from the largest to the smallest spheroid. We can also deduce this result from equation (6) for the equilibrium values. The parameter *a* scales the constant *u* in that equation, lowering the graph of the quartic and raising the value of the positive root. However, Freyer also reports that the basic growth rates of the cell lines (either in spheroids or monoculture) did not correlate with final spheroid size. However, there could be an interplay of parameters at work to mask such a correlation. Altering other parameters may give a similar result. The model offered here at least shows the possibility of a positive correlation between final size and thickness of the proliferating cell layer.

**Figure 5:**
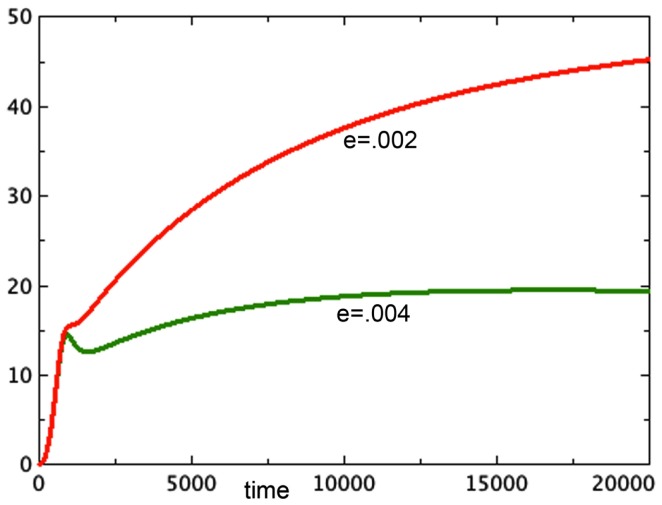
**Two runs are shown with different rates of necrosis: *e_Q,N_* = 0.002 (top) and *e_Q,N_* = 0.004 (bottom)**. *a* = 0.01, *b_P,Q_* = 0.005, *c_Q,P_* = 0.005, *d* = 0.0002, *f* = 0.001, *h* = 0, *m* = 0.0001, *P*_0_ = 0.01, *Q*_0_ = 0.01, *N*_0_ = 0.

A second observation of Freyer is that spheroid equilibrium size is correlated with the size of the spheroid at the onset of necrosis. His paper shows data in which the spheroid size at the onset of necrosis is estimated from data and the eventual spheroid size is inferred by fitting data to a Gompertz curve. This observation is thus more of an expectation based on models than an actual pair of measurements. Nonetheless, we ask whether Model 3E in this paper can support this expectation. In continuous models such as this one, there is an instantaneous start of necrosis, although the quantity may be quite small. If one spheroid exhibits the start of necrosis at a larger size than another, it could be due to different rates of transition from the quiescent to the necrotic pool, described by parameter *e* in our model. The moment at which necrosis becomes visible in the spheroid would be earlier for the model with the higher value of *e*. In Figure 5, we see the growth of two versions of Model 3E with different values of the parameter *e*. This difference does indeed produce spheroids of different sizes. The spheroid that has the higher rate of necrosis is the smaller one, consistent with Freyer’s observations.

**Figure 6:**
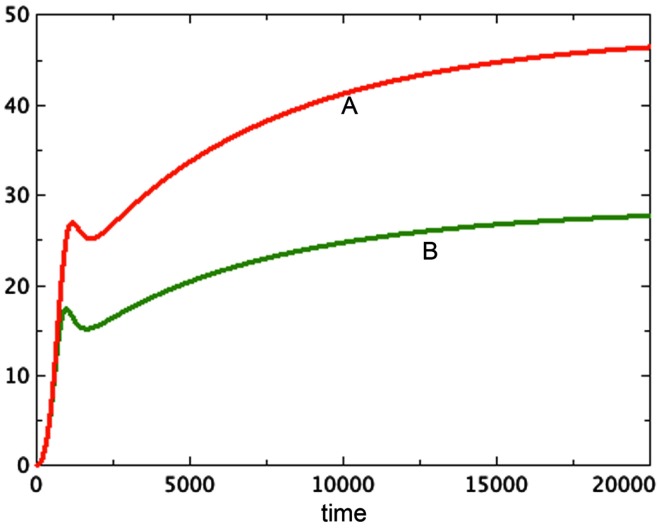
**Here we see two graphs of total tumor size**. The larger, A, is a single spheroid in isolation. The smaller, B, is two graphs superimposed of identical spheroids grown together as in Figure 5, where the necrosis factor of both spheroids affects each system. *a* = 0.01, *b_P,Q_* = 0.005, *c_Q,P_* = 0.005, *d* = 0.0002, *e_Q,N_* = 0.002, *f* = 0.001, *h* = 0, *m* = 0.0001, *P*_0_ = 0.01, *Q*_0_ = 0.01, *N*_0_ = 0.

The possibility that tumor necrosis factor affects not only the spheroid producing it, but also others in the same flask, also helps explain the observation (Folkman and Hochberg, 1973) that the average size of spheroids in a flask was inversely proportional to the number of spheroids in the flask. In Figure 6 we see the comparison of two systems: one represents a system with two identical spheroids. However, the necrosis factor used was the sum of the necrosis of both spheroids. That is, both spheroids suffer the effect of all of the toxin in the combined system. These two grow identically and reach a terminal size of approximately 27 units at *t* = 20,000. A third spheroid is grown in isolation, with the same initial conditions. It gets much larger, reaching about 45 units at *t* = 20,000. Thus neighboring spheroids limit each others’ growth, creating the result observed by Folkman and Hochberg (1973).

## 4 Discussion

We have shown that a simple model with compartments representing proliferating, quiescent and necrotic cells, can explain a variety of observations on the growth and development of tumor spheroids *in vitro*. The successful model includes a growth term proportional to surface area, and a death term for proliferating cells that depends on the amount of tumor necrosis present. It also includes linear transitions between the proliferating and quiescent pools, and between the quiescent and necrotic pools. It includes a linear term for dissolution of necrotic cells as well. With these few ingredients we have a system that produces spheroids that eventually develop three layers of proliferating, quiescent and necrotic cells. Growth of these spheroids eventually stops. When the growth stops, there remains a thin layer of actively proliferating cells at the boundary. Under some assumptions about what might create spheroids of different sizes, we see that the final size of the spheroids is correlated with thickness of the proliferating shell. Under the assumption of differing rates of necrosis we see spheroids of different terminal sizes, corresponding to a later appearance of visible necrosis. Under the hypothesis that multiple spheroids in the same flask share tumor necrosis factor with each other, we have a model in which the more spheroids are in a flask, the smaller the average size when growth ends. In short, the model we have selected fits a variety of qualitative observations about tumor spheroid growth.

Although more complex than the Gompertz model, the model presented here is not so complex that it becomes computationally unfeasible to match it to the development of a given tumor spheroid. It would be useful to modelers to have some data sets that track *P*, *Q*, and *N* over time for several cell lines. A model such as the one presented here will only be of practical use if it is tuned to a specific type of cell. The potential then exists for building more realistic models of *in vivo* tumors that are based on parameters solidly gained from the simpler *in vitro* spheroid.

**Figure 7:**
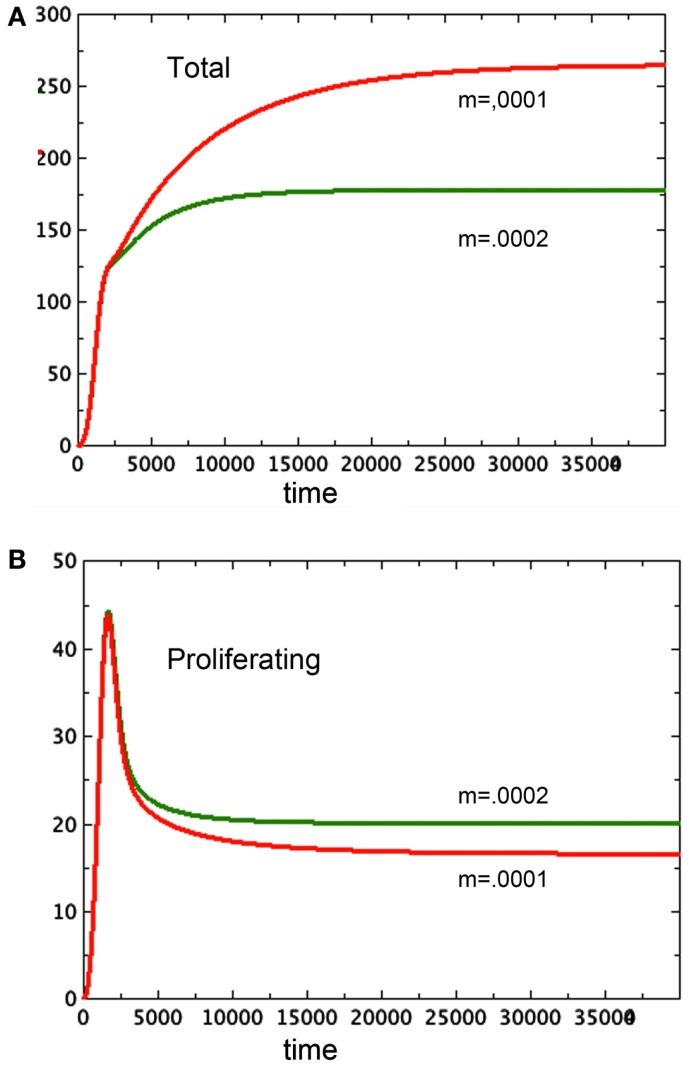
**Here we see two graphs of total tumor size in 7A and size of the proliferating pool, 7B**. The larger spheroid in 7A has the lower removal rate of *N*, set at *m* = 0.0001. The smaller spheroid has *m* = 0.0002. Note the reversal of size of the proliferating component of the spheroid. *a* = 0.01, *b_P,Q_* = 0.005, *c_Q,P_* = 0.005, *d* = 0.0002, *e_Q,N_* = 0.002, *f* = 0.0001, *h* = 0, *P*_0_ = 0.01, *Q*_0_ = 0.01, *N*_0_ = 0.

Tumor spheroids are supposed to be a reasonable proxy for tumors *in vivo* before the onset of angiogenesis. However, existing models of angiogenesis do not take tumor necrosis factor into account, even though it affects the growth of the proliferating cells. Using a model such as the one developed here as the basis for a more complex one that includes angiogenesis has the potential to illuminate one function of circulation currently left out of these models: the ability of blood flow to remove toxins. Figure 7 elucidates this observation. Here we see two spheroid models that are identical except for one small change: *N*, along with the toxin it represents, is being removed from one of them at a steady rate of 0.0001*N* in one of them and 0.0002*N* in the other. The spheroid with the greater clearance rate of necrosis has a smaller total size, but it has a greater quantity of proliferating tissue. This observation adds to the complexity of angiogenesis.

One purpose of this study is eventually to develop a simple model incorporating both necrosis and angiogenesis. With data on the time development of both plate and spheroid cultures of a given cell type one, could easily find best fit parameters for model 3E as well as a few of the others. A model that is a good approximation of spheroid behavior may be extended by coupling it with a simple model of angiogenesis, as in Hahnfeldt et al. (1999), Komorova and Mironov (2005), or with a version of a more complex model as in Stamper et al. (2007) that has been reduced via a sensitivity analysis (as in Wallace and Winsor, 2012) to a simpler situation. One would do this be extending the spheroid model in two ways. First, the growth term would be replaced by an expression approximating contact area between proliferating cells and nutrient supply. Second, the clearance of necrosis factor would become a function of contact between necrotic tissue and blood supply. Numerous papers have explored the nature of the contact regions between tumor and nutrient through the development of the geometry of both tumor and vasculature (as in Sansone et al., 2001), competition among cell types (as in Scalerandi et al., 2001), and other features. A simple model, however, might approximate the situation through mutual dependence of blood supply, volumes of the three quantities discussed here, and a fixed or evolving fractal dimension of contact. A model with few parameters may then be fitted to data sets for various cell lines to give a crude characterization of growth properties for cell types that goes beyond the basic growth rate determined from plate culture.

Hirschhaeuser et al. (2010) survey the uses of *in vitro* spheroids to study the interaction of tumors with their microenvironment, including various therapies. They point out the potential role of spheroids in selecting the most promising interventions at an early, and less expensive, stage of research. Mathematical models of spheroids that extend the simple model presented in this paper to include therapies or other interactions would be useful for selecting optimal delivery protocols to be tested *in vivo* as therapeutic interventions. Models *in silico* allow quick exploration of the result of variations in timing and dosage of therapies. Studies of cancer therapies *in vivo* could be doubly informed by *in vitro* spheroid studies that suggest which therapies work and why, combined with *in silico* models suggesting best strategies for delivery.

## Conflict of Interest Statement

The authors declare that the research was conducted in the absence of any commercial or financial relationships that could be construed as a potential conflict of interest.
